# Comparison of Substrate Specificity of *Escherichia Coli p*-Aminobenzoyl-Glutamate Hydrolase with *Pseudomonas* Carboxypeptidase G

**DOI:** 10.4236/aer.2014.21004

**Published:** 2014-03

**Authors:** Cassandra M. Larimer, Dejan Slavnic, Lenore D. Pitstick, Jacalyn M. Green

**Affiliations:** Department of Biochemistry, Chicago College of Osteopathic Medicine, Midwestern University, Downers Grove, IL, USA

**Keywords:** *p*-Aminobenzoyl-Glutamate Hydrolase, Carboxypeptidase G, Folate Catabolism

## Abstract

Reduced folic acid derivatives support biosynthesis of DNA, RNA and amino acids in bacteria as well as in eukaryotes, including humans. While the genes and steps for bacterial folic acid synthesis are known, those associated with folic acid catabolism are not well understood. A folate catabolite found in both humans and bacteria is *p*-aminobenzoyl-glutamate (PABA-GLU). The enzyme *p*-aminobenzoyl-glutamate hydrolase (PGH) breaks down PABA-GLU and is part of an apparent operon, the *abg* region, in *E. coli*. The subunits of PGH possess sequence and catalytic similarities to carboxypeptidase enzymes from *Pseudomonas* species. A comparison of the subunit sequences and activity of PGH, relative to carboxypeptidase enzymes, may lead to a better understanding of bacterial physiology and pathway evolution. We first compared the amino acid sequences of AbgA, AbgB and carboxypeptidase G_2_ from *Pseudomonas sp.* RS-16, which has been crystallized. Then we compared the enzyme activities of *E. coli* PGH and commercially available *Pseudomonas* carboxypeptidase G using spectrophotometric assays measuring cleavage of PABA-GLU, folate, aminopterin, methotrexate, 5-formyltetrahydrofolate, and 5-methyltetrahydrofolate. The *K_m_* and *V*_max_ values for the folate and anti-folate substrates of PGH could not be determined, because the instrument reached its limit before the enzyme was saturated. Therefore, activity of PGH was compared to the activity of CPG, or normalized to PABA-GLU (nmole/min/µg). Relative to its activity with 10 µM PABA-GLU (100%), PGH cleaved glutamate from methotrexate (48%), aminopterin (45%) and folate (9%). Reduced folates leucovorin (5-formyltetrahydrofolate) and 5-methyltetrahydrofolate were not cleaved by PGH. Our data suggest that *E. coli* PGH is specific for PABA-GLU as its activity with natural folates (folate, 5-methyltetrahydrofolate, and leucovorin) was very poor. It does, however, have some ability to cleave anti-folates which may have clinical applications in treatment of chemotherapy overdose.

## 1. Introduction

Reduced folates and their various one-carbon derivatives are essential for growth and cell division of plants, animals, and bacteria. Humans cannot synthesize this vitamin and therefore require folate in their diet, in contrast to plants and bacteria which can make folate [1]. This makes the folate biosynthetic pathway an ideal target for antibiotics. Indeed, the sulfa drugs, which were among the first antibiotics, inhibit the folate biosynthetic pathway. Later, it was found that the antibiotic trimethoprim inhibited dihydrofolate reductase, a different part of the folate pathway. While antibiotic resistance has developed in response to each of these, together these two drugs are still commonly and successfully used in treatment of bacterial infections.

**Figure 1:**
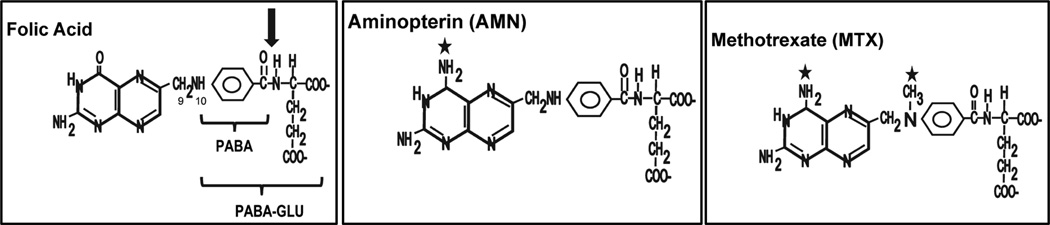
Structures of folic acid, aminopterin and methotrexate. The arrow indicates the point of cleavage. The stars indicate the points where aminopterin and methotrexate differ from folic acid.

The study of the folate biosynthetic pathway has been fruitful, leading to development of a variety of drugs, both antibacterial and chemotherapeutic. In contrast, our understanding of the intracellular catabolism of folic acid in bacteria remains incomplete [2]. In 1956 Nickerson and Webb demonstrated that *E. coli* incubated with the folate analog aminopterin displayed an initial growth inhibition followed by a recovery [3] [4]. Further analysis revealed that the cells produced folate breakdown products *p*-aminobenzoyl-glutamate (PABA-GLU) and pteridine, demonstrating cleavage at the C_9_-N_10_ bond (Figure 1). There have also been many studies describing purification and characterization of various carboxypeptidases with varying abilities to cleave the terminal glutamate of folates and related analogs; these have largely been isolated from *Pseudomonas* [5]-[9], or *Flavobacterium* [10] [11], respectively. These enzymes have historically been of clinical interest owing to their multiple practical applications, particularly for their use in generating intermediates for chemical synthesis. Specificities for various folyl and anti-folyl substrates among these enzymes varied, however, as did their metal dependence. Most enzymes required divalent zinc. Of these enzymes, carboxypeptidase G_2_ (CPG_2_) from *Pseudomonas* sp. RS-16 has been both the most well-studied and the most clinically useful of the folate-degrading enzymes thus studied. Known clinically as glucarpidase (Voraxaze^®^; BTG International, Inc.), CPG_2_, a zinc-dependent enzyme, is used intravenously for treatment of patients with toxic levels of blood methotrexate, an anti-folate used for chemotherapy [12].

**Figure 2:**
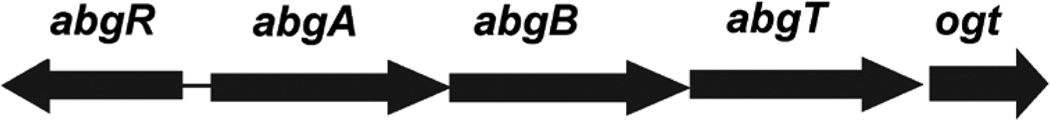
The *abg* region of *E. coli*.

Our laboratory studies an area of the *E. coli* genome that appears to be an operon encoding proteins involved in metabolism of folate breakdown product PABA-GLU (Figures 1, 2). Very little was known about gene organization and folate catabolism in bacteria until the *abg* region was identified in *E. coli* [13]. AbgT imports PABA-GLU [14], while *abgA* and *abgB* encode subunits of the heterotetrameric *p-*aminobenzoyl-glutamate hydrolase (PGH) [15]. This study also demonstrated that PGH required divalent manganese for activity, and failed to cleave a range of dipeptides.

The current research was performed to expand our knowledge of the catalytic properties of PGH and to compare its sequence and properties with the carboxypeptidases. The amino acid sequences of AbgA and AbgB were used in BLAST searches, and were compared to the most well characterized *Pseudomonas* carboxypeptidase, *Pseudomonas sp* RS-16 carboxypeptidase G_2_ (*Ps.*CPG_2_). We also tested the ability of PGH to cleave a variety of folate and anti-folate substrates, and compared its activity to commercially available carboxypeptidase G. These findings will help determine the physiological role of PGH and the other bacterial carboxypeptidases, as well as identify enzymatic activities that may have clinical applications.

## 2. Materials and Methods

### 2.1. Materials

Folic acid, *p-*aminobenzoyl-glutamic acid, and purified *Pseudomonas* Carboxypeptidase G (CPG) were obtained from Sigma-Aldrich (St. Louis, MO). Methotrexate, aminopterin, 5-formyltetrahydrofolate, and 5-methyltetrahydrofolate were obtained from Schircks Laboratories (Switzerland). The Bio-Rad protein assay kit was obtained from Bio-Rad (Hercules, CA). The Ni-nitrilotriacetic acid (NTA) agarose used for protein purification was from Qiagen (Valencia, CA). SDS-PAGE gels (7.5%) were obtained from Bio-Rad (Hercules, CA). *E. coli* PGH was isolated from *E. coli* as previously described [15].

### 2.2. Protein Sequence Alignments

Sequence alignments were performed using the free online software created and supported by the UniProt Consortium [16]. The amino acid sequences for *E. coli* AbgA (P77357), *E. coli* AbgB (P76052), and *Pseudomonas* sp. RS-16 carboxypeptidase G_2_ (P06621) were obtained from the UniProt website.

### 2.3. Enzyme Assays

Continuous spectrophotometric assays were used to measure the ability of CPG and PGH to utilize a variety of folates and anti-folates as substrates, as previously described [5] [8]. Cleavage of glutamate from the following substrates was monitored using the following wavelengths and extinction coefficients: folate (303 nm; 8000 M^−1^∙cm^−1^); aminopterin (310 nm; 6000 M^−1^∙cm^−1^); methotrexate (320 nm; 8,300 M^−1^∙cm^−1^); 5-formyltetrahydrofolate (308 nm; 3300 M^−1^∙cm^−1^); 5-methyltetrahydrofolate (312 nm; 5000 M^−1^∙cm^−1^). All enzyme and protein assays were performed in triplicate, and a control lacking enzyme was included.

The assay for cleavage of PABA-GLU was performed essentially as described previously [15]. Reaction mixtures consisted of 50 mM Tris, pH 8.5, 10 mM *β*-mercaptoethanol, 5 mM MnCl_2_, and varying concentrations of PABA-GLU. Assays for cleavage of PABA-GLU by CPG were performed similarly, except that ZnCl_2_ (2 mM) was substituted for the MnCl_2_. Assays were initiated with 50 µg of either *Pseudomonas* CPG or purified *E. coli* PGH and incubated at 37°C. Samples (0.5 mL) were taken with time (30, 60, 90 and 120 sec) and terminated with addition of 1.0 M sodium citrate, pH 5.5 (0.5 mL). Product *p*-aminobenzoate (PABA) was extracted into 2 mL of ethyl acetate, and absorbance of the ethyl acetate layer containing PABA was measured at 284 nm (*ε* = 13,400 M^−1^∙cm^−1^ [15]). The velocity was calculated as the slope of the line (nmoles of product versus time in minutes).

### 2.4. Determination of the Glutamate Yield from Cleavage of Various Substrates by HPLC

Enzyme reaction mixtures were analyzed for glutamate content using an HPLC-based method, as described previously [15]. The procedure involved removal of PGH or CPG with a filter device, followed by modification of an aliquot of reaction mixture by dabsylation. The resulting dabsylated products were subjected to HPLC analysis. The amounts of glutamate generated as product in the reaction mixtures were measured by comparison to a standard curve generated by a series of control samples containing a range of glutamate concentrations. Samples were performed in triplicate.

### 2.5. Data Analysis

Statistical analysis was performed using GraphPad Prism 5 (GraphPad Software, Inc, San Diego, CA).

## 3. Results

### 3.1. Amino Acid Sequence Alignment

**Figure 3:**
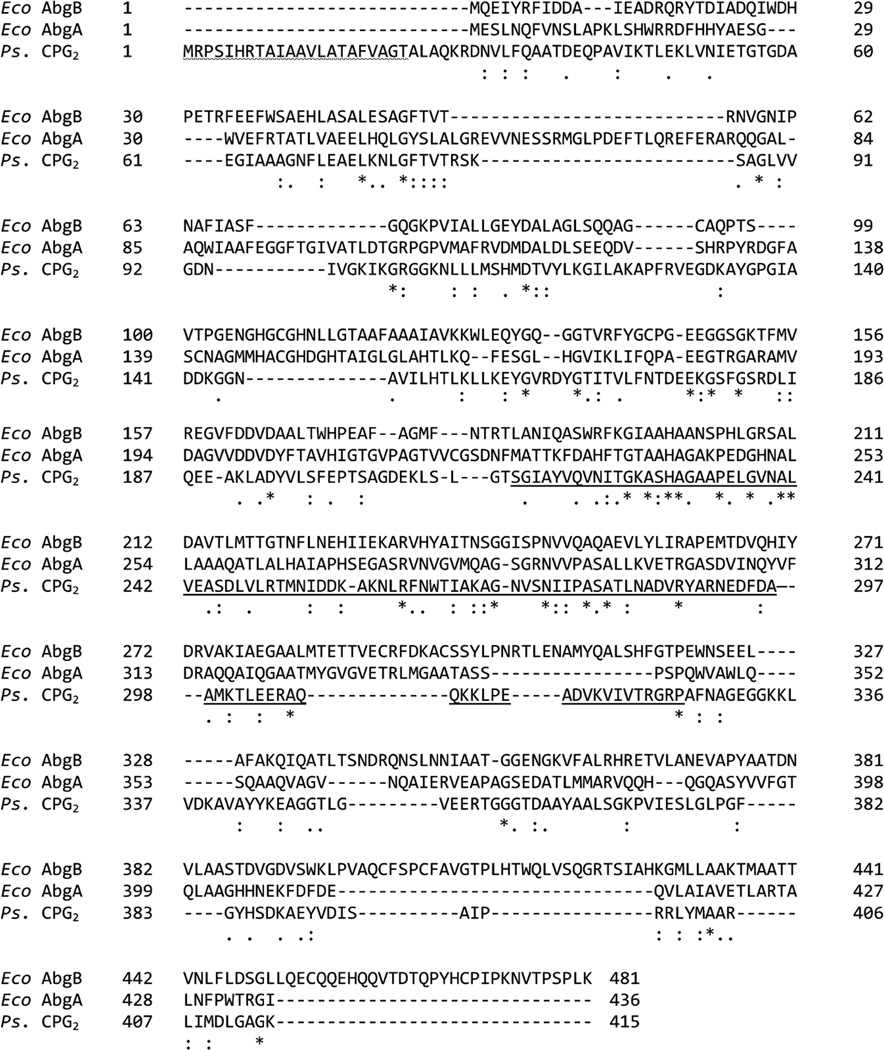
Alignment of the amino acid sequence of *E. coli* AbgB and AbgA, and *Ps.* CPG_2_. *Ps.* CPG_2_ and each subunit of *E. coli* PGH were aligned using UniProt. The signal peptide is indicated by wavy underlining at the N-terminus of CPG_2_. Identical amino acid residues are indicated by a star (*), strongly similar residues are indicated by a colon (:), and weakly similar groups are indicated by a period (.). The residues associated with subunit interactions in CPG_2_ are underlined.

BLAST searches were performed using the amino acid sequence of both AbgA and AbgB, respectively, and all proteins that were identified as homologous were from bacteria, suggesting that PGH is only found in prokaryotes (data not shown). The sequences of *E. coli* AbgB and AbgA were compared to, and aligned with, carboxypeptidase G_2_ from *Pseudomonas* sp. RS-16 (*Ps*-CPG_2_; Figure 3). When first cloned and sequenced, AbgA and AbgB were found to be similar to one another [13]; they are 18.9% identical with 140 similar positions between AbgA (436 amino acids; 46.6 kDa) and AbgB (481 amino acids; 52.2 kDa). AbgA and AbgB are 18.4% and 15.5% identical to *Ps*-CPG_2_, respectively, with similarity at 120 and 128 positions, respectively. The crystal structure of *Ps*-CPG_2_ has been resolved, enabling identification of amino acid residues involved in catalysis, metal binding, and subunit interaction [17]. *Ps*-CPG_2_ holoenzyme is a homodimer; each subunit is composed of 415 amino acids (41.8 kDa) arranged in two domains. The larger, catalytic domain possesses two zinc cations in close proximity at the active site while the smaller domain is involved in subunit interactions. While we theorized that each PGH subunit might perform one of the functions of *Ps*-CPG_2_, *i.e.*, subunit interaction or catalytic function, the homology among AbgB, AbgA and Ps-CPG2 spans the entire length of the proteins.

Both *Ps*-CPG_2_ and PGH are metalloproteins. *Ps*-CPG_2_ is zinc(II) dependent, however, while PGH requires divalent manganese for activity. We repeated the amino acid alignments of AbgA and AbgB individually with PS-CPG_2_ in order to better compare the sequence motifs involved in metal binding; these data are summarized in Table 1. The crystal structure of *Ps*-CPG_2_ shows that each zinc ion is coordinated by three groups on the protein: a histidine, an aspartate and a glutamate. One aspartic acid (D141) coordinates both zinc ions, as each carboxylate oxygen coordinates with a different zinc ion. In the sequence homologous to that binding zinc(1), in both AbgA and AbgB the glutamate remains conserved. The histidine is also conserved in AbgA, but in AbgB this residue is a glycine. At the position of the aspartic acid in *Ps*-CPG_2_ there is a histidine in AbgB and in AbgA (H146); however, in AbgA there is an aspartic acid immediately adjacent (D147). Thus, AbgA is homologous for 2 – 3 of the zinc(1) metal binding residues, while AbgB is homologous for only one. For the second zinc, *Ps*-CPG_2_ and AbgB/AbgA are similar but some of the amino acids have exchanged positions. At the homologous position of *Ps*-CPG_2_ Asp141, both AbgA and AbgB have a histidine, while at the position of the zinc2-coordinated histidine in *Ps*-CPG_2_, AbgA and AbgB possess an aspartate and a glutamate, respectively. In summary, there are many parallels among the metal-binding sequence motifs of *Ps*-CPG_2_, AbgA and AbgB, but they are not perfectly aligned. These differences may correspond to differences between divalent zinc and manganese as cofactors.

There are key structural differences between PGH and *Ps*-CPG_2_. PGH iscomposed of two non-identical subunits in a heterotetramer, while*Ps*-CPG_2_ is a homodimer [15] [18]. AbgA and AbgB associate strongly enough that isolation of PGH is achieved with a polyhistidine tag on the carboxyl terminus of the AbgB subunit alone, enabling purification using a nickel metal affinity chromatography system [15]. Rowsell *et al.* identified a dimer interface domain in *Ps*-CPG_2_ consisting of amino acid residues 214 – 325 [17]. They suggested that hydrophobic interactions and hydrogen bonding both contribute to the association of the *Ps*-CPG_2_ subunits. The similarity/ identity in this region between *Ps*-CPG_2_ and the *abg* proteins is consistent with AbgA and AbgB forming a dimer together, which then associates with another dimer to form the tetramer that predominates when analyzed by size exclusion chromatography/mass spectrometry [15]. In addition, *Ps*-CPG_2_ is found in the periplasmic space, and is targeted to that cellular compartment by its 22 amino acid N-terminal leader sequence [19]. This leader sequence is absent in the *abg* genes.

### 3.2. Cleavage of Oxidized Folate Analogs and PABA-GLU by PGH and CPG

Prior studies demonstrated that *E.coli* PGH cleaves folate catabolite PABA-GLU; however, its ability to cleave folate (1 mM) was extremely poor [15]. The prior experiment involved a time consuming system in which samples of reaction mixtures were filtered to remove enzyme, then subjected to chemical modification (dabsylation) of the product glutamate which was then analyzed by HPLC. While this method demonstrated that folate and a variety of dipeptides were poor substrates, it was not suitable for kinetic experiments.

In order to further characterize PGH with regard to its ability to cleave a variety of reduced and oxidized folates and related structures, we used continuous spectrophotometric assays developed previously for enzymatic cleavage of methotrexate, aminopterin, folate, leucovorin, and 5-methyltetrahydrofolate [8]. We compared the activity of PGH with *Pseudomonas* CPG for several reasons. First, CPG would serve as a positive control to assure that the assay was working properly. Secondly, while *Ps.* CPG_2_ is used clinically as Glucarpidase (Voraxaze^®^, BTG International, Inc.), and while we could not obtain this enzyme, *Pseudomonas* CPG (Sigma) is likely very closely related.

**Figure 4:**
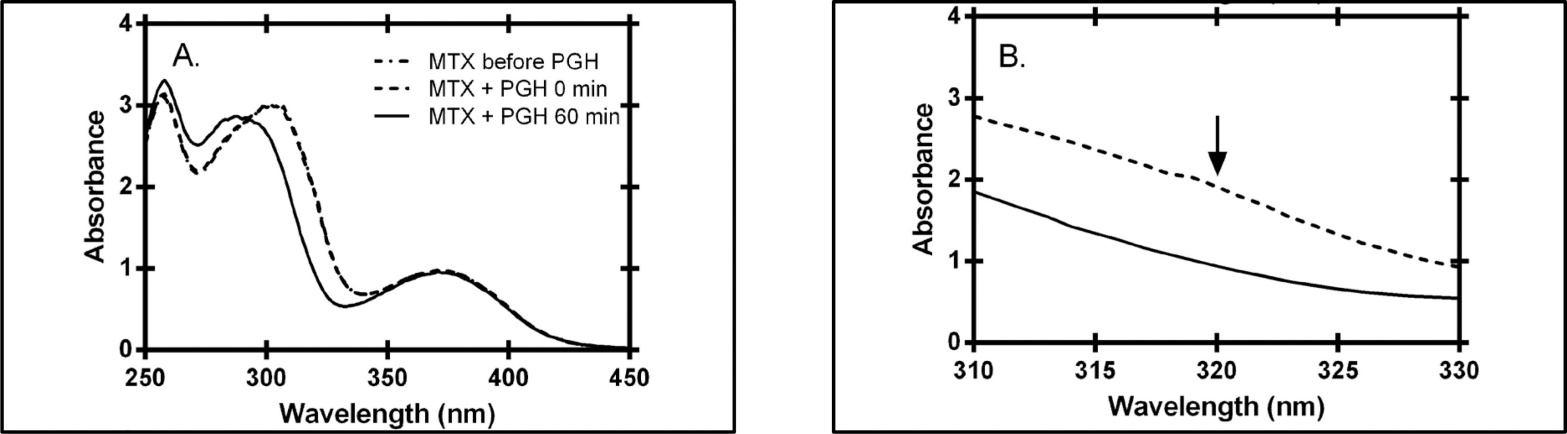
Changes in absorbance associated with cleavage of methotrexate (MTX) by PGH. Panel A. Wavelength scans of MTX before (dotted line) and after (solid line) reaction with PGH. The reaction mixture included 50 mM Tris buffer, pH 7.3, 1 mM MnCl_2_, and 100 µM MTX. A wavelength scan was taken before and immediately after addition of enzyme; these two scans were indistinguishable. The reaction was initiated with the addition of PGH (44 µg). The reaction mixture was incubated for one hour at room temperature, and then the final scan was taken. Panel B. Difference spectrum, magnified between 310 nm and 330 nm. The point of maximal difference, which was used for continuous assays of MTX cleavage, in indicated by an arrow.

**Figure 5:**
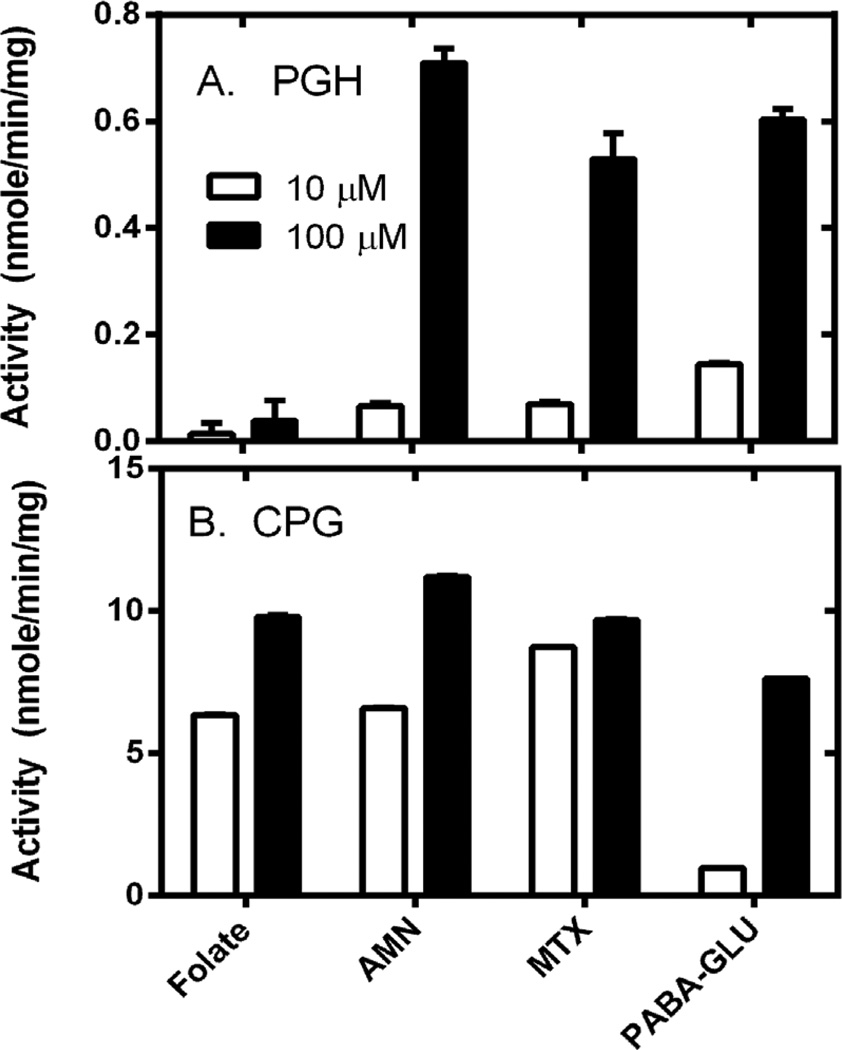
Enzyme activity observed using 10 µM or 100 µM folate, aminopterin, methotrexate, or PABA-GLU using (A) PGH (B) CPG. Enzyme reactions were performed as described in the methods. Briefly reaction mixtures consisted of 50 mM Tris, pH 8.5, substrate (10 µM or 100 µM) and divalent cation (1 mM MnCl_2_ for PGH and 1 mM ZnCl_2_ for CPG). Reactions were initiated with addition of enzyme, and the change in absorbance was monitored over time for 5 minutes. The initial rate was determined by linear regression analysis of data using Graph Pad Prism 5. Samples were done in triplicate. Data are reported as average ± standard deviation.

Spectrophotometric assays have been developed for the cleavage of glutamate from both reduced and oxidized forms of folates, as well as the oxidized form of the anti-folates. These assays are based on the difference spectra between the intact substrate and the product formed when glutamate is released (Figure 4). The molar absorptivity coefficients for the difference spectra for folic acid, aminopterin and methotrexate range between 6000 – 83,000 M^−1^∙cm^−1^. The molar absorptivity coefficients for the parent compounds, however, range from ~22,000 to 27,000 M^−1^∙cm^−1^; thus, solutions of these compounds greater than ~100 µM possess a peak absorbance beyond the limit of most spectrophotometers. Consequently we were unable to observe sufficient saturation of PGH to measure either a *K_m_* and *V*_max_ value for folate, aminopterin or methotrexate (data not shown). Therefore we chose two concentrations, 10 µM and 100 µM, and compared the ability of 50 µg of CPG and PGH, respectively, to catalyze cleavage of each substrate: folate, aminopterin and methotrexate (Figure 5).

PGH and CPG exhibit some major differences in their enzymatic activities, as evidenced by the data in Figure 5. First, if one examines the y-axis scale for each enzyme, which is greater than ten-fold higher for CPG than for PGH, it is clear that in general PGH is a much less active enzyme than CPG. Compared to CPG, PGH displayed only a very weak ability to cleave folic acid. Aminopterin, methotrexate and PABA-GLU were much better substrates for PGH than folic acid. CPG is a less discriminating enzyme, as it utilized all of these substrates relatively well. The comparison of the activity for 10 µM versus 100 µM substrates also yielded information about these enzymes. PGH displayed an almost linear increase in activity when the substrate concentration for aminopterin and methotrexate was increased between 10 µM and 100 µM. This indicates that the *K_m_* values for these substrates are relatively high for PGH, *i.e.* PGH is not saturated for substrate. In contrast, the activity for CPG with 10 µM versus 100 µM folate, aminopterin, and methotrexate was similar for each substrate; the observation that CPG was approaching saturation even at 10 µM substrate indicates a low *K_m_* value for that substrate. The only substrate for which CPG demonstrates little saturation, with an increase in activity between 10 and 100 µM substrate and therefore a high *K_m_* value, is PABA-GLU.

### 3.3. Cleavage of Reduced Folate by PGH and CPG

Next we measured the ability of PGH to cleave several physiologically relevant reduced folates, in comparison with CPG. The primary circulating form of folate in the human body is the monoglutamate form of 5-methyltetrahydrofolate [20]. Leucovorin, also referred to as citrovorum factor, folinic acid, or 5-formyltetrahydrofolate, is a natural form of reduced folate found inside cells. It is also used clinically as a treatment for elevated levels of methotrexate in the blood [21]. As shown in Table 2, CPG demonstrates higher activity with these substrates when compared to PABA-GLU; PGH was unable to cleave these reduced folates as observed by no change in absorbance when incubated with enzyme. The negative results with PGH were confirmed with HPLC analysis of reaction mixtures analyzed by HPLC as described in the methods (data not shown).

## 4. Discussion

It is surprising that research into pathways of folate catabolism have not been as thoroughly studied as those of folate biosynthesis, given how knowledge of unique biosynthetic pathways has led to many useful compounds, ranging from antibiotics, chemotherapeutic agents, and herbicides. One reason folate catabolism has been less studied than biosynthesis may be the assumption that it results from the simple oxidative degradation of reduced folates, independent of enzymatic catalysis [2]. Indeed, in 1957 Blakely demonstrated that reduced folates were particularly susceptible to inactivation by air [22]; later studies confirmed that spontaneous breakdown of reduced folates upon exposure to air involved oxidative cleavage at the N_9_-N_10_ bond, releasing PABA-GLU [23]. Early studies however also supported the notion that folate catabolizing enzymes existed. Wood and Hitchings studied the transport and breakdown of folates and anti-folates by several bacteria, and found that uptake of folate required glucose, implying that it was an active process [24]; several bacterial strains degraded folate, producing concomitant amounts of PABA-GLU.

Efforts to purify a mammalian enzyme that cleaved the N_9_-N_10_ bond of 5-formyltetrahydrofolate surprisingly led to the purification of rat ferritin [25]. Each ferritin molecule catalyzed a single turnover *in vitro*. The case for physiological significance was strengthened by the observation that increased expression of rat ferritin in a cell culture system correlated with both increased folate turnover and decreased intracellular folate levels. While it is unclear if this is the only mode whereby folate is enzymatically degraded, it is accepted that PABA-GLU is a product of folate catabolism in humans; whether this is primarily by oxidative (non-enzymatic) processes or also by enzyme catalysis [26]. PABA-GLU, as well as its metabolite acetamido-PABA-GLU (a product of hepatic metabolism of PABA-GLU), are in urine and in the gastrointestinal tract where endogenous bacteria, include *E. coli*, reside. These compounds are used routinely as markers of folate turnover [27] [28].

Very little was known about gene organization of folate catabolizing genes in bacteria until the *abg* region was identified in *E. coli* (Figure 2). While we have demonstrated that AbgT imports PABA-GLU, and PGH cleaves it, the physiological significance of this is not clear. PABA-GLU is not a physiologically relevant substrate for the biosynthetic pathway as the kinetics of dihydropteroate synthase are not favorable [29]. It is possible, however, that this operon participates as a recycling or salvage pathway for *p*-aminobenzoate (PABA) which is an intermediate in biosynthesis of folate. Biosynthesis of PABA is synthesized from the action of two enzymes encoded by three genes, *pabA, pabB*, and *pabC*; the former two genes encode 4-amino-4-deoxychorismate synthase, which competes with enzymes involved in aromatic amino acid biosynthesis for the intermediate chorismate. Under cellular conditions of nutrient deprivation, the salvage of PABA from partially degraded folates may relieve folate deficiency.

CPG enzymes in general are able to catalyze glutamate cleavage from a broad range of folate and anti-folate substrates, while PGH has been shown here to be much more specific for PABA-GLU. Widely found in nature, *Pseudomonas* secrete a variety of enzymes, which make them a major tool in bioremediation [30]. In contrast, *E. coli* cells live primarily in the mammalian gut. Unlike CPG, PGH is not secreted. Production of enzymes that can non-specifically degrade a range of reduced and oxidized folates would be expected to be very toxic to the cell, so it is reasonable that *E. coli* generates an enzyme that is more specific than CPG. A comparison of the amino acid sequences of AbgA and AbgB, the subunits of PGH, with the sequence and crystal structure of *Ps*-CPG_2_ offers some clues with regard to the differences in substrate specificity [17]. The crystal structure of *Ps*-CPG_2_ was determined in the presence of metal ion but in the absence of folate substrates, however, so specific predictions regarding folate substrate binding motifs remain difficult. One can predict that the binding site for PGH is smaller than that of *Ps*-CPG_2_, as PABA-GLU lacks the pteridine moiety when compared to the folates and anti-folates favored as substrates by *Ps*-CPG_2_ (Figure 1). The difference in metal requirement for each enzyme may contribute to the differences in substrate specificity as well. *Ps*-CPG_2_ is a homodimer where each subunit contains a dinuclear zinc site. While the number of metal binding sites for each subunit of PGH has not been determined, purified PGH, an *α*_2_*β*_2_ tetramer, is inactive until Mn^2+^ has been provided, and enzyme activity with other divalent cations is minimal [15]. In *Ps*-CPG_2_ a single aspartate (D141) coordinates both zinc ions in the active site, and this amino acid is not conserved in either AbgA or AbgB, suggesting that there are differences in the metal binding sites of these subunits. Also, Mn^2+^ has a radius of 0.75 Angstrom while Zn^2+^ is smaller, with a radius of 0.65 Angstrom [31]; thus, the metal may contribute to steric factors hindering activity with substrates larger than PABA-GLU in the binding site of PGH.

## 5. Conclusion

In summary, we have compared the sequence and activity of PGH from *E. coli* with carboxypeptidase enzymes from *Pseudomonas*. PGH displays much more specificity for its ability to cleave folate catabolite PABA-GLU when compared to *Pseudomonas* CPG, which cleaves a wide range of both reduced and oxidized folates and folate analogs. We hypothesize that this is a mechanism enabling salvage of oxidized intracellular folates, while protecting against degradation of reduced folates. We are continuing our studies of the *abg* region to better understand the role of these genes and proteins in *Escherichia coli* physiology.

## Figures and Tables

**Table 1:** Comparison of *Ps.* CPG_2_ metal binding sites with *E. coli* AbgA and AbgB homologies, based on alignments performed with *Ps.* CPG_2_ and each subunit alone.

*Ps*-CPG_2_	*E. coli* AbgA homolog	*E. coli* AbgB homolog
Zinc1/2*: Asp (D141)	His(H146); Asp (D147)	His (H111)
Zinc1: Glu (E176)	Glu (E183)	Glu (E146)
Zinc1: His(H385)	His (H406)	Gly (G409)
Zinc2: Glu (E200)	Thr(T205)	His (H171)
Zinc2: His (H112)	Asp (D116)	Glu (E81)

* In *Ps*-CPG_2_ each carboxylate oxygen of Asp (D141) coordinates with a different zinc.

**Table 2:** Comparison of activity of PGH and CPG with reduced folates and PABA-GLU.

Substrate (10 µM)	PGH (nmole/min/mg)	PGH %	CPG (nmole/min/mg)	CPG %
PABA-GLU	0.145 + 0.0022	100	0.972 + 0.0035	100
5-formyl-tetrahydrofolate	ND	0	1.49 + 0.00013	150%
5-methyl-tetrahydrofolate	ND	0	4.47 + 0.00026	460%

Enzyme reactions were performed as described in the methods. Briefly, reaction mixtures consisted of 50 mM Tris, pH 7.3, substrate (10 µM) and divalent cation (1 mM MnCl_2_ for PGH and 1 mM ZnCl_2_ for CPG). Reactions were initiated with addition of enzyme, and the change in absorbance was monitored over time for 5 minutes. The initial rate was determined by linear regression analysis of data using Graph Pad Prism 5. Samples were done in triplicate. Data are reported as average ± standard deviation. ND means not detected.
